# Women Empowerment and Its Relation with Health Seeking Behavior in Bangladesh

**Published:** 2015-06

**Authors:** AKM Mainuddin, Housne Ara Begum, Lal B. Rawal, Anwar Islam, SM Shariful Islam

**Affiliations:** 1 International Center for Diarrheal Disease Research, Dhaka, Bangladesh; 2 Institute of Health Economics, University of Dhaka, Bangladesh; 3 International Center for Diarrheal Disease Research, Dhaka, Bangladesh AND School of Health Policy & Management, Faculty of Health, York University, Toronto, Canada; 4 International Center for Diarrheal Disease Research, Dhaka, Bangladesh AND Center for International Health, Ludwig Maximilian University of Munich, Germany

**Keywords:** Women empowerment, mobility, decision making, health seeking behavior

## Abstract

**Objective:** Over the last few decades, Bangladesh has made significant progress towards achieving targets for the Millennium Development Goals (MDGs) and women empowerment. This study is aimed at identifying the levels and patterns of women empowerment in relation to health seeking behavior in Bangladesh.

**Materials and methods:** We conducted a cross-sectional study among 200 rural married women in Cox’s Bazar district in Bangladesh using multi stage sampling technique and face-to-face interview. Data was collected on socio-economic characteristics, proxy indicators for women empowerment in mobility and health seeking behavior related decision making. Bivariate and multivariate regression analyses were performed to identify associations between women empowerment in relation to health seeking behavior on mobility and decision making, controlling the effect of other independent variables.

**Results:** The results showed that only 12% women were empowered to decide on their own about seeking healthcare and 8.5% in healthcare seeking for their children. In multivariate analysis women empowerment in health seeking behavior was higher among age group 25-34 years (OR 1.76, [CI = 0.82-3.21]), women’s education, husband’s education, age at marriage > 18 years (OR 6.38, [CI = 0.98-4.21]) and women’s working status (OR 16.44, [CI = 0.79-2.71]).

**Conclusion:** Women empowerment enhances their decision-making authority regarding health seeking behavior. Acknowledging and adopting the implications of these findings are essential for an integrated health and development strategy for Bangladesh and achieving the MDGs.

## Introduction

In recent years Bangladesh has made significant progress in reducing maternal and child mortality and is on track for achieving the targets of MDG 4 and 5 (1). At the same time, the country also progressed in terms of women empowerment (2). Considering its fundamental value in improving women’s well-being and overall positive impact on the family (3) , women empowerment is considered as an important and essential public policy goal. It has been argued that economically empowered women can play a more active role in household decision-making and have greater bargaining power to increase spending on education and health (4). Women empowerment expands the freedom of choice and action to shape women’s lives (5) and in the long run not only contributes to individual woman, but to the family, society and the country as a whole. Women empowerment is considered as necessary condition for development, although it is not a sufficient condition (6). Women empowerment has several dimensional focuses and envisages greater access to knowledge, social and economic resources, and greater participation in economic and political decision making processes (7). It seeks change in the sexual division of labor, equal access to food, healthcare, education, employment opportunities, ownership of land and other assets and access to the media. Despite the involvement in numerous household and income generating activities women’s contribution to the family income is yet to be recognized equally to that of men (Islam). Evidence shows that women lag behind men in many aspects of development such as educational attainment, employment, social and political power and exposure to the media (8). Bangladesh ranks 100 out of 128 countries in terms of gender equality as reported by the Global Gender Gap Index 2007 developed by the World Economic Forum (9). 

Evidence shows that women empowerment has a profound influence on the use of health services that could be linked to reproductive health outcomes (10). A number of studies also suggest that women empowerment could increase contraceptive use (11- 14). A few studies have examined other dimensions of women’s empowerment, including decision making regarding household economy and family size, unhindered mobility within and outside the community, freedom from coercion or violence by the spouse and/or other family members, women’s political and legal awareness, and their participation in public protests and political campaigning (15, 16). However, the relationship between women’s empowerment and health is multi-dimensional, and complex. A better understanding and appreciating these complex relationships between women’s empowerment and health are critical for developing effective health strategies for a country like Bangladesh. Unfortunately few studies have extensively explored this relationship in context of developing countries. The aim of this study is to identify the levels and differentials in women’s empowerment in terms of women’s mobility and decision making authority in health seeking behavior in a rural area of Bangladesh. The results will help better understand the relationship between women empowerment and health seeking behavior of rural women in Bangladesh and could be used to design more integrated and effective gender development policies and programs for health.

## Materials and methods

Participants and Settings: A cross-sectional study was carried out among 200 married rural women at Cox's Bazar district. Study site covers 8 sub-districts, 3 municipalities, 67 union parishads and 975 villages located in southern Bangladesh on the edge of the Bay of Bengal (www.mycoxsbazar.info). Applying a two-stage sampling technique one sub-district (Sadar Thana or main sub-district) was selected from 8 sub-districts. At the second stage, four villages were randomly selected as the study area. From each village 50 participants were conveniently selected and interviewed. 220 women were approached for the interview with almost 91% response rate. 

Data collection: A pre-tested semi-structured questionnaire was used to collect data. Data was collected on socio-economic characteristics of the participants, proxy indicators for women empowerment in mobility and health seeking behaviour related decision making. Eight graduate female interviewers were recruited locally and trained to interview the participants with necessary conceptual and contextual understanding. The interviewers carried out one to one interview at participant’s house maintaining proper confidentiality. The interviewers cross checked the questionnaire for missing data and inconsistency after the interview. To provide necessary conceptual and systematic support, two experienced supervisors monitored the interview and cross checked the data regularly.

Ethics: Following the spirit of the Helsinki Declaration written informed consent was obtained from each participant and their right to refuse taking part in the study or withdraw at any point was explained to them. The goals and objectives of the study were fully explained the interviewees. The study was approved by the Ethical Review Board of the Department of Health Economics, University of Dhaka, Bangladesh. 

Measurements: The study used women’s empowerment as a dependent variable and measured it in terms of women’s mobility and decision making authority. Women’s mobility consisted of three indicators such as mobility to shop alone, mobility to go to another village alone and mobility to visit the hospital and/or a health centre alone. In the Bangladesh Demographic and Health Survey (8), women’s freedom of movement is measured by one indicator - whether women can go to hospital. The responses consisted of three categories (1. goes alone, 2. goes with other and 3. cannot go). In this study, as noted earlier, three indicators were used, each with two categories (1 = yes and 2 = no). The second component of women’s empowerment consisted of five indicators such as women’s decision making power in own healthcare, decision making power in child healthcare, decision making power in large household purchases, decision making power in choosing daily food items and finally decision making power in visiting a relative’s home. Each of the dependent variables had two categories “yes” and “no”. To better understand the differentials in women’s empowerment in terms of women’s mobility and decision making authority, different socio-economic, demographic and cultural factors were used to examine the effect on each of the dependent variables 

Data analysis: Data was subjected to univariate, bivariate and multivariate analyses as appropriate. At univariate stage, frequency and percentage/proportion were distributed against the participant’s independent (socio-economic, demographic, cultural characteristics) and dependent variables (women’s mobility and decision making authority) (not shown). At the bivariate stage, logistic regression analyses were carried out for each of the outcome variables (three in women’s mobility and three in decision making authority) by each of the independent variables without controlling the effect of other independent variables (presented in Table 1). At the last stage, multivariate logistic regression analyses were carried out to better understand the relationship between the dependent and the independent variables. The effect of all the independent variables was observed on each of the dependent variables. However, effect of other independent variables was controlled so that the relationship between the dependent and the independent variables is not confounded. All analyses were performed using SPSS version 16 statistical software (SPSS Corporation, Chicago, IL, USA). Statistical tests were two-sided; P values < 0.05 were considered statistically significant.

## Results

A total of 19% of the participants were aged 24 years old followed by 30%, 28% and 23% aged 25-34 years, 35-44 years and ≥ 45 years respectively. Almost all the participants (95.0%) had some years of formal education. A total of 52.5% had 1-5 years education while 32.5% and 10% had 6-10 years and > 10 years education respectively. Of the participants 55.5% had some sorts of formal work and 44.5% were housewives. About half (50.5%) of the respondents were Muslims; while 30.5% were Buddhists, 11.5%, Hindus and 7.5% Christians. About one-third (30.5%) women were members of any NGO’s. An overwhelming majority of the participants had some years of formal education (93%), were married for ≥ 18 years (81.0%) and 55% of women talked about family planning with their husband. Around one-third of the women (32.0%) enjoyed freedom in shopping alone, 27.5% in going to another village independently and 21% in visiting hospital for treatment alone. However, it is apparent that women had less empowerment in decision making authority than in mobility. Only 12.0% had empowerment in deciding own healthcare seeking, 8.5% in healthcare seeking for their children, 11.5% in spending resources on major household items, 14.5% in choosing daily food items and 7.5% in visiting relative’s homes.

The results of bivariate analysis for women’s empowerment in mobility and in decision making are presented in Table 1. Husband’s education (1-5 years) had significant odds (OR = 14.244, p < 0.05) for women’s empowerment in mobility to shop alone. Interestingly women’s empowerment in shopping alone seems to be significantly associated with their empowerment in working, discussing family planning with their husbands, deciding to seek heath care for their children, deciding major household purchases and deciding daily food items Besides, women’s working status, empowerment in deciding own healthcare seeking, healthcare for children, major household purchase, and visit relative’s homes had significant association with women’s going to another village alone.

**Table 1:** Results of bivariate logistic regression analysis for women’s empowerment in mobility and decision making

Variables	Empowerment in Mobility			Empowerment in Decision Making				
	MSA	MGAVA	MVHA	EOHS	ECHS	EPLHI	EPDFI	EVRH
	OR	OR	OR	OR	OR	OR	OR	OR
**Participant’s age **								
** ≤ 24 (RC)**	**1.000**	**1.000**	**1.000**	**1.000**	**1.000**	**1.000**	**1.000**	**1.000**
** 25-34 years**	**0.748**	**0.432**	**0.783**	**1.246**	**0.571**	**0.559**	**0.540**	**0.242**
** 35-44 years**	**0.396**	**0.430**	**0.225** *	**0.107** **	**0.407**	**1.529**	**0.585**	**0.158**
** ≥ 45 years**	**0.887**	**0.988**	**0.771** *	**0.271** *	**0.969**	**0.891**	**0.488** *	**0.115**
**Participant’s education**								
** No education (RC)**	**1.000**	**1.000**	**1.000**	**1.000**	**1.000**	**1.000**	**1.000**	**1.000**
** 1-5 years**	**2.193**	**1.820**	**0.572**	**0.220**	**0.160**	**0.882**	**11.000** *	**2.667**
** 6-10 years**	**2.419**	**2.744**	**0.279**	**0.108**	**0.033**	**0.220** *	**0.362** *	**0.200** *
** >10 years**	**3.075**	**2.762**	**0.968**	**0.218**	**0.040** **	**0.424**	**0.715**	**0.127** *
**Participant’s religion **								
** Islam (RC)**	**1.000**	**1.000**	**1.000**	**1.000**	**1.000**	**1.000**	**1.000**	**1.000**
** Hinduism**	**1.360**	**4.144** **	**0.651**	**2.405**	**1.094**	**1.741**	**3.383** *	**1.416**
** Christianity**	**1.200**	**16.390** ***	**3.401**	**1.369**	**0.000**	**2.100**	**0.476**	**0.092**
** Buddhism**	**2.916**	**1.589**	**0.341**	**0.335**	**0.131**	**4.424**	**5.657** ***	**0.175**
**Husband’s education **								
** No education (RC)**	**1.000**	**1.000**	**1.000**	**1.000**	**1.000**	**1.000**	**1.000**	**1.000**
** 1-5 years**	**14.244** *	**1.549**	**0.439**	**0.189**	**0.000**	**0.891**	**0.404** ***	**0.286**
** 6-10 years**	**3.100**	**2.183**	**0.190**	**0.935**	**0.096**	**0.778**	**0.212** ***	**0.151** **
** > 10 years**	**1.009**	**3.708**	**0.116**	**0.115**	**0.077**	**0.445** *	**0.102** ***	**0.088** ***
**Age at marriage **								
** < 18 years (RC)**	**1.000**	**1.000**	**1.000**	**1.000**	**1.000**	**1.000**	**1.000**	**1.000**
** ≥ 18 years)**	**0.838**	**0.462**	**0.395**	**7.471** **	**2.125**	**1.212**	**1.262**	**1.570**
**NGO membership **								
** No (RC)**	**1.000**	**1.000**	**1.000**	**1.000**	**1.000**	**1.000**	**1.000**	**1.000**
** Yes**	**0.828**	**1.134**	**0.899**	**0.425**	**0.815**	**0.770**	**0.839**	**0.882**
**Working status **								
** No (RC)**	**1.000**	**1.000**	**1.000**	**1.000**	**1.000**	**1.000**	**1.000**	**1.000**
** Yes**	**2.771** *	**5.353** **	**33.298** ***	**2.106**	**3.096**	**0.094**	**3.832** ***	**1.558**
**Discussing with husband of FP**								
** No (RC)**	**1.000**	**1.000**	**1.000**	**1.000**	**1.000**	**1.000**	**1.000**	**1.000**
** Yes**	**3.220** **	**1.257**	**1.961**	**2.178**	**2.107**	**0.105** ***	**5.248** ***	**1.268**
**MSA**								
** Not shopped (RC)**	**N/U**	**N/U**	**N/U**	**1.000**	**1.000**	**1.000**	**1.000**	**1.000**
** Shopped alone**	**N/U**	**N/U**	**N/U**	**0.939**	**0.279**	**4.898** ***	**1.619**	**0.437**
**MGAV**								
** Not went (RC)**	**N/U**	**N/U**	**N/U**	**1.000**	**1.000**	**1.000**	**1.000**	**1.000**
** Went alone**	**N/U**	**N/U**	**N/U**	**0.259** *	**0.044** ***	**8.088** ***	**0.369**	**0.509**
**MVHA **								
** Not went (RC)**	**N/U**	**N/U**	**N/U**	**1.000**	**1.000**	**1.000**	**1.000**	**1.000**
** Went**	**N/U**	**N/U**	**N/U**	**0.266** *	**2.009**	**8.968** ***	**0.729**	**0.298** **
**EHS**								
** Not decided (RC)**	**1.000**	**1.000**	**1.000**	**N/U**	**N/U**	**N/U**	**N/U**	**N/U**
** Decided**	**0.441**	**0.168** ***	**0.087** ***	**N/U**	**N/U**	**N/U**	**N/U**	**N/U**
**ECHS**								
** Not decided (RC)**	**1.000**	**1.000**	**1.000**	**N/U**	**N/U**	**N/U**	**N/U**	**N/U**
** Decided**	**2.128** **	**2.145** ***	**1.983** ***	**N/U**	**N/U**	**N/U**	**N/U**	**N/U**
**WPLHI**								
** Not decided (RC)**	**1.000**	**1.000**	**1.000**	**N/U**	**N/U**	**N/U**	**N/U**	**N/U**
** Decided**	**1.348** *	**1.463** **	**1.874** ***	**N/U**	**N/U**	**N/U**	**N/U**	**N/U**
**EPDFI**								
** Not decided (RC)**	**1.000**	**1.000**	**1.000**	**N/U**	**N/U**	**N/U**	**N/U**	**N/U**
** Decided**	**0.451** *	**0.145** *	**0.411**	**N/U**	**N/U**	**N/U**	**N/U**	**N/U**
**EVRH**								
** Not decided (RC)**	**1.000**	**1.000**	**1.000**	**N/U**	**N/U**	**N/U**	**N/U**	**N/U**
** Decided**	**0.607**	**0.298** **	**0.677**	**N/U**	**N/U**	**N/U**	**N/U**	**N/U**

(MSA = mobility in shopping alone, MGAV = mobility in going another village, MVHA = mobility in visiting hospital alone, EHS = empowerment in own healthcare seeking, ECHS = empowerment in children’s healthcare seeking, EPLHI = empowerment in purchasing large household items, EPDFI = empowerment in daily food items purchasing and EVRH = empowerment in visiting relative’s home)

(RC) = Reference Category, N/U = not used in the analyses,

*** p <0.001;

** p<0.01;

* p < 0.05

Significant association was found between women’s empowerment in deciding daily food items and women’s age (≥ 45 years), education (1-5 years and 6-10 years), religion, husband’s education, working status and discussion with husband about family planning. Regarding women’s empowerment in deciding to visit relative’s home, significant associations were found with women’s education (6-10 years and > 10 years), husband’s education (6-10 years and > 10 years) and empowerment in mobility to visit hospital. 

The Multivariate regression analysis revealed that there were differences in terms of significant variables, level of significance compared to that of bivariate analysis (Table 2). Husband’s education was no longer significant with women’s empowerment in mobility to shop alone (presented in Table 2). Buddhist women were significantly more (OR = 6.496, p < 0.05) likely to shop alone. Women empowered in deciding major household purchases and daily food items were also significantly more likely to be empowered in mobility in shopping alone. Similar to bivariate analysis, Christian women appeared to be more likely (OR = 11.88, p < 0.05) to go to another village alone compared to Muslim. Working women, women empowered with deciding major household purchases, daily food items and deciding to visit relative’s home appeared to affect women’s empowerment in mobility to go another village significantly. Women’s religion (Buddhism), working status, women empowered in deciding major household purchases and daily food items appeared significant with women’s mobility in visiting hospital. 

After controlling the effect of other independent variables, age group 35-44 years were significantly less likely (OR = 0.01, p < 0.05) to be empowered in deciding about own healthcare seeking. Husband’s education (1-5 years), age at marriage and NGO membership also had significant impact on women’s decision making about own healthcare seeking. The significant impact on women’s empowerment in deciding major household purchases appeared with women’s age (25-3 4 years), education (6-10 years), religion (Christianity and Buddhism), husband’s education (> 10 years), NGO membership and women’s empowerment in mobility to go another village. For women empowerment in deciding daily food items, the significant impact appeared with women’s age (≥ 45 years), education (1-5 years, 6-10 years and > 10 years), religion (Buddhism), husband’s education (1-5 years, 6-10 years and > 10 years) and discussion with husband about family planning.

## Discussion

In this study we identified the levels and patterns of women empowerment in relation to health seeking behavior in a rural area of Cox’s Bazar district. Approximately one-third participants reported going to shop alone, one-fourth going to another village alone and one-fifth going to hospital alone. The mobility to go to health center in our study is remarkably low compared to national level where 65.8% had mobility in going to health center (8). A small number of participants in our study reported empowerment in deciding own healthcare, healthcare for children, deciding major household purchasing, deciding daily food items and visiting relative’s home respectively. A study conducted by NIPORT in 2007, reported 13.8% women decided own healthcare, 18.7 children’s healthcare, 8.5 for major household purchases, 32.6 for daily food items and 12.6% for visiting relative’s home (8). These findings also suggest that women in Cox’s Bazar are lagging behind to a greater amount from the other regions in this county in terms of women’s mobility and empowerment. 

Different socio-economic and cultural factors affect women’s mobility and decision making patterns. Women’s aged > 24 years were significantly less empowered in deciding own healthcare and visiting relative’s home compared to women 35-44 years, in deciding daily food items compared to women ≥ 45 years. An exception was deciding major household purchases which had higher odds compared to women aged ≤ 24 years. It is likely that women ≤ 24 years are women with higher education, higher knowledge on modern goods, more aware of the women’s rights due to being later generation and hence more likely to come over many of the cultural barriers and traditional roles of women. On the other hand, women aged > 24 years are more likely to maintain the traditional gender roles between husband and wife and less likely to consider extra familial demands even if it becomes own health matters. The exception may be because in rural areas, major household items are rarely purchased.

**Table 2:** Results of multivariate logistic regression analyses of women’s empowerment in mobility and decision making

Variables	Empowerment in Mobility			Empowerment in Decision Making				
	MSA	MGAVA	MVHA	EHS	ECHS	EPLHI	EPDFI	EVRH
	OR	OR	OR	OR	OR	OR	OR	OR
**Participant’s age **								
** ≤ 24 (RC)**	**1.000**	**1.000**	**1.000**	**1.000**	**1.000**	**1.000**	**1.000**	**1.000**
** 25-34 years**	**4.899**	**0.067**	**0.394**	**1.764** *	**0.126**	**8.073** *	**0.773**	**0.407**
** 35-44 years**	**0.963**	**0.132**	**0.961**	**0.011** ***	**0.507**	**9.753**	**0.541**	**0.040** *
** ≥ 45 years**	**2.052**	**0.502**	**1.432**	**0.224**	**0.199**	**2.062**	**0.273** *	**0.112**
**Participant’s education**								
** No education (RC)**	**1.000**	**1.000**	**1.000**	**1.000**	**1.000**	**1.000**	**1.000**	**1.000**
** 1-5 years**	**1.252**	**1.519**	**1.134**	**1.413**	**0.693**	**0.793**	**0.006** **	**1.307**
** 6-10 years**	**1.574**	**1.804**	**1.963**	**1.818**	**0.008** *	**0.109** *	**0.008** **	**1.327**
** > 10 years**	**2.489**	**2.171**	**2.312**	**2.179**	**0.021**	**0.312**	**0.010** ***	**2.452**
**Participant’s religion **								
** Islam (RC)**	**1.000**	**1.000**	**1.000**	**1.000**	**1.000**	**1.000**	**1.000**	**1.000**
** Hinduism**	**2.411**	**2.868**	**0.197**	**1.889**	**0.120**	**1.989**	**3.442**	**0.009** **
** Christianity**	**0.939**	**11.880** *	**1.072**	**0.525**	**0.000**	**18.558** *	**0.610**	**0.009** **
** Buddhism**	**6.496** **	**0.865**	**0.097** **	**0.128**	**0.034**	**10.657** ***	**20.046** ***	**0.001** ***
**Husband’s education **								
** No education (RC)**	**1.000**	**1.000**	**1.000**	**1.000**	**1.000**	**1.000**	**1.000**	**1.000**
** 1-5 years**	**1.530**	**1.250**	**1.577**	**30.648** *	**0.005**	**0.562**	**0.004** ***	**0.245**
** 6-10 years**	**0.280**	**1.282**	**1.885**	**3.116**	**0.012**	**0.634**	**0.002** ***	**0.856**
** > 10 years**	**0.193**	**2.190**	**2.329**	**3.581**	**0.006** *	**0.556** *	**0.005** ***	**0.115**
**Age at marriage**								
** < 18 years (RC)**	**1.000**	**1.000**	**1.000**	**1.000**	**1.000**	**1.000**	**1.000**	**1.000**
** ≥ 18 years)**	**0.648**	**2.952**	**6.376** *	**0.037** **	**0.909**	**0.406**	**1.264**	**7.053**
**NGO membership **								
** Not member (RC)**	**1.000**	**1.000**	**1.000**	**1.000**	**1.000**	**1.000**	**1.000**	**1.000**
** Member**	**0.500**	**0.853**	**0.818**	**0.203** *	**0.849**	**4.018** *	**1.341**	**1.019**
**Working status **								
** Not working (RC)**	**1.000**	**1.000**	**1.000**	**1.000**	**1.000**	**1.000**	**1.000**	**1.000**
** Working**	**1.633**	**30.610** ***	**16.443** **	**1.045**	**1.579**	**0.163**	**1.442**	**1.636**
**Discussion with husband about family planning**								
** Not discussed (RC)**	**1.000**	**1.000**	**1.000**	**1.000**	**1.000**	**1.000**	**1.000**	**1.000**
** Discussed**	**1.751**	**1.983**	**1.626**	**1.486**	**1.093**	**1.493**	**5.248** ***	**1.320**
**MSA**								
** Not shopped (RC)**	**N/U**	**N/U**	**N/U**	**1.000**	**1.000**	**1.000**	**1.000**	**1.000**
** Shopped alone**	**N/U**	**N/U**	**N/U**	**2.572**	**0.176**	**0.176**	**0.770**	**0.246**
**MGAV**								
** Not went (RC)**	**N/U**	**N/U**	**N/U**	**1.000**	**1.000**	**1.000**	**1.000**	**1.000**
** Went alone**	**N/U**	**N/U**	**N/U**	**0.635**	**0.024** **	**0.024** **	**1.423**	**0.943**
**MVHA**								
** Not went (RC)**	**N/U**	**N/U**	**N/U**	**1.000**	**1.000**	**1.000**	**1.000**	**1.000**
** Went**	**N/U**	**N/U**	**N/U**	**0.564**	**8.367**	**8.367**	**0.976**	**0.212**
**EHS**								
** Not decided (RC)**	**1.000**	**1.000**	**1.000**	**N/U**	**N/U**	**N/U**	**N/U**	**N/U**
** Decided**	**0.400**	**0.403**	**0.265**	**N/U**	**N/U**	**N/U**	**N/U**	**N/U**
**ECHS**								
** Not decided (RC)**	**1.000**	**1.000**	**1.000**	**N/U**	**N/U**	**N/U**	**N/U**	**N/U**
** Decided**	**1.748**	**0.790**	**0.858**	**N/U**	**N/U**	**N/U**	**N/U**	**N/U**
**WPLHI**								
** Not decided (RC)**	**1.000**	**1.000**	**1.000**	**N/U**	**N/U**	**N/U**	**N/U**	**N/U**
** Decided**	**2.462** *	**2.122** **	**1.740** *	**N/U**	**N/U**	**N/U**	**N/U**	**N/U**
**EPDFI**								
** Not decided (RC)**	**1.000**	**1.000**	**1.000**	**N/U**	**N/U**	**N/U**	**N/U**	**N/U**
** Decided**	**1.472** **	**1.234** ***	**2.190** *	**N/U**	**N/U**	**N/U**	**N/U**	**N/U**
**EVRH**								
** Not decided (RC)**	**1.000**	**1.000**	**1.000**	**N/U**	**N/U**	**N/U**	**N/U**	**N/U**
** Decided**	**0.328**	**0.150** ^*^	**0.858**	**N/U**	**N/U**	**N/U**	**N/U**	**N/U**

(MSA = mobility in shopping alone, MGAV = mobility in going another village, MVHA=mobility in visiting hospital alone, EHS = empowerment in own healthcare seeking, ECHS = empowerment in children’s healthcare seeking, EPLHI = empowerment in purchasing large household items, EPDFI=empowerment in daily food items purchasing and EVRH=empowerment in visiting relative’s home)

RC = Reference Category; N/U = Not used in the analyses;

*** p < 0.001;

** p < 0.01;

* p < 0.05

When a family purchases major household items (furniture, television, etc.), it is likely that it has often long term demand from women. In most of the cases, women with > 24 years of age had less mobility but the result was not statistically significant. 

Women’s education had significant impact on women’s empowerment (deciding child healthcare, purchasing large household items and daily food items) but not on women’s mobility. But the findings of this study is inconsistent with other studies where it has been argued that schooling for girls and enrolment rates reduce gender inequality in education (17, 18). But the less empowerment with schooling in this study may be because of women’s lower education may not contribute to the attitudinal as well as behavioral changes among women within the traditional family settings. Almost similarly, husband’s education had significant impact only on women’s empowerment. Women whose husband’s had some years of formal education were less empowered in terms of decision making for child health care, major household purchases and daily food items except higher empowerment in deciding own healthcare seeking. It is likely that rural educated husbands are more self-confidant making them more authoritative than non-educated husbands. Also this may be because of relational construct of empowerment in the sense that women’s performance depends on men’s response, even being educated, in the family settings and men in power that serves only men’s interest. 

Religion had significant impact on women’s mobility and empowerment. Christian and Buddhist women had greater mobility and empowerment compared to Muslim women. This may be may be more because of fundamental practices of Islam in the region than religion itself. According to (19) ‘Purdah’ and segregation of sexes in the Muslim society limit women’s physical mobility and contacts by making them subordinate of the male members of the family. Consequently, decisions mostly come from male partner of the household. However, the findings also revealed that women affiliated with other religions were less likely to decide to visit relative’s home. 

This study found that women married at less than 18 years had higher mobility (except mobility in shopping alone) while less empowerment (except decision making authority in daily food items and visiting relative’s home). However, statistical significance was found only in empowerment in deciding own healthcare. It is likely that women married at ≥ 18 years are also likely to have better education and have higher likelihood of outgoing behavior due to delayed marriage. However, in traditional society of Bangladesh women are dominated by permeated with patriarchal values especially after marriage. Moreover, in order to exercise control on women, having higher mobility, it is likely that male partner poses extra pressure so that women cannot exercise empowerment in the family setting. 

Working women had higher mobility and empowerment, but results were statistically significant only with women’s mobility in going to another village and visiting hospitals. Women employment is considered an important determinant of women empowerment (6, 20). Most probably working women are more competitive while competing roles give women greater excess to extra familiar sources of information and resources with increasing their potential autonomy in family settings. 

Despite significant impact of NGO membership on women’s own healthcare seeking and purchasing major household items, opposed to the expectation, women involved with NGO had lower mobility and decision making authority to some extent. Studies found that participation in credit programs is positively associated with a woman's level of empowerment defined as a function of her relative physical mobility, economic security, ability to make various purchases on her own, freedom from domination and violence within the family, political and legal awareness, and participation in public protests and political campaigning (21). NGOs are considered to frame many activities for women empowerment (22). The reasons behind the lower empowerment in mobility and decision making in our study may be women’s double standard when they are within the NGO and when they are at home, women’s incapacity to change one’s behavior within the patriarchal family settings and lack of encouragement by family members. 

Women deciding about major household purchases and daily food items had higher mobility in terms of shopping, going another village alone and visiting hospitals. This finding is, to some extent, consistent with another study that found that women who are more mobile have higher odds of being empowered (23). It is expected because women’s empowerment in decision making reflects the acceptance of women’s views in the family and reliance on women for household needs. Women who discussed about FP with husband had higher mobility, empowerment and are more likely to decide about daily food items. It is likely that these women are valued by their husband, have higher convincing power, and have higher role to play in the family. 

Women’s mobility had significant impact on women empowerment. Women who went alone to another village had significant lower empowerment in terms of deciding child healthcare and large household purchases. The reason for this negative impact may be that women in rural areas are usually brought up with an environment where from the childhood they accustomed to move agricultural field to supply food to the father and to some extent due to moving to another village for education. These findings also may indicate the lower resource of women, and hence lower capacity in decision making. 

This study has several limitations. First, this study was carried out on the conveniently selected sample population in one district of Bangladesh and the findings cannot be generalized. Second, only three indicators were used to measure women’s mobility and empowerment in decision making. Thus, the findings do no indicate women’s overall mobility and empowerment but rather specifically focus on these indicators. Finally, the study participants were only women. Information from both men and women could generate more reliable information on women’s mobility and empowerment.

## Conclusion

The finding of this study shows that women empowerment increases their health seeking behavior and should be recognized as a norm and an important component in health systems design. Religious misperception should be removed so that women can move outside the home along with increased participation in working. Media campaign and inclusion of religious leaders at the local level may help to disseminate messages for women rights and empowerment. Measures should be taken to increase education capacities for both men and women and ensure that no woman marry before age 18 years. Women’s workforce participation should be increased so that women can be self-dependent financially. Finally, consideration should be given on male participation not only in maternal healthcare service utilization but also in every aspect of conjugal life so that a culture of discussion between husband and wife about the family matters can be ensured. The implications of these findings are essential for an integrated health and development strategy for Bangladesh for achieving the MDGs.

## References

[1] DHS (2011). Demographic and Health Survey.

[2] Chant SH (2007). Gender, generation and poverty: exploring the feminisation of poverty in Africa, Asia and Latin America.

[3] Meinzen-Dick R, Pandolfelli L, Dohrn S, Athens J (2005). Gender and collective action: A conceptual framework for analysis. International Research Workshop on Gender and Collective Action.

[4] Woods N (2008). Whose aid? Whose influence? China, emerging donors and the silent revolution in development assistance. International Affairs.

[5] (2005). Mosedale S: Assessing women's empowerment: towards a conceptual framework. Journal of International Development.

[6] Faridi MZ, Chaudhry IS, Anwar M (2009). The socio-economic and demographic determinants of women work participation in Pakistan: evidence from Bahawalpur District. A Research Journal of South Asian Studies.

[7] Kabeer N (2005). Gender equality and women's empowerment. A critical analysis of the third millennium development goal 1. Gender & Development.

[8] Koenig MA, Jamil K, Streatfield PK, Saha T, Al-Sabir A, Arifeen SE (2007). Maternal health and care-seeking behavior in Bangladesh: findings from a national survey. International Family Planning Perspectives.

[9] Hausmann R, Tyson LD, Zahidi S (2007). The global gender gap index 2007. The global gender gap report.

[10] Blanc AK (2001). The effect of power in sexual relationships on sexual and reproductive health: an examination of the evidence. Studies in family planning.

[11] Harvey SM, Bird ST, De Rosa CJ, Montgomery SB, Rohrbach LA (2003). Sexual decision making and safer sex behavior among young female injection drug users and female partners of IDUs. Journal of sex research.

[12] Pulerwitz J, Gortmaker SL, DeJong W (2000). Measuring sexual relationship power in HIV/STD research. Sex Roles.

[13] Wang RH, Chiou CJ (2008). Relative contribution of intrapersonal and partner factors to contraceptive behavior among Taiwanese female adolescents. Journal of Nursing Scholarship.

[14] Wingood GM, DiClemente RJ (2000). Application of the theory of gender and power to examine HIV-related exposures, risk factors, and effective interventions for women. Health education & behavior.

[15] Ghuman SJ, Lee HJ, Smith HL (2006). Measurement of womenâ€™s autonomy according to women and their husbands: Results from five Asian countries. Social Science Research.

[16] Schuler SR, Hashemi SM (1994). Credit programs, women's empowerment, and contraceptive use in rural Bangladesh. Studies in family planning.

[17] Sathar ZA, Kazi S (2000). Women's autonomy in the context of rural Pakistan. The Pakistan Development Review.

[18] Chaudhry IS, Faridi MZ, Anjum S (2010). The Effects of Health and Education on Female Earnings: Empirical Evidence from District Vehari. Pakistan Journal of Social Sciences.

[19] Mernissi F (1987). Beyond the veil: Male-female dynamics in modern Muslim society. Indiana University Press.

[20] Ashraf J, Ashraf B (1993). Estimating the gender wage gap in Rawalpindi city. The Journal of Development Studies.

[21] Husain Z, Mukerjee D, Dutta M (1996). Are women self-help group members economically more empowered in left-run municipalities?. Development in Practice.

[22] Haider SKU (2013). The Impacts of NGOs on the Socio-Economic Situation of the Poor: A Case Study in Rajshahi City, Bangladesh. International Journal of Community Development.

[23] Samman E, Santos ME (2009). Agency and Empowerment: A review of concepts, indicators and empirical evidence.

